# Multicultural Psychological Empowerment Scale for Saudi Women

**DOI:** 10.3389/fpsyg.2021.768616

**Published:** 2022-01-27

**Authors:** Hanaa Faize A. Moubarak, Asyraf Afthanorhan, Eisa Sneitan N. Alrasheedi

**Affiliations:** ^1^Program of Social Work, Department of Social Sciences, College of Arts, University of Ha’il, Ha’il, Kingdom of Saudi Arabia; ^2^Faculty of Business and Management, Universiti Sultan Zainal Abidin, Kuala Nerus, Malaysia; ^3^Department of English, College of Arts, University of Ha’il, Ha’il, Kingdom of Saudi Arabia

**Keywords:** empowerment, women empowerment, psychological empowerment, meaningfulness, impact, self-efficacy, self-determination

## Abstract

The current study aimed to construct a multicultural psychological empowerment scale for Saudi women depending on the four dimensions of psychological empowerment, namely, meaningfulness, impact, self-efficacy, and self-determination. It was applied to a sample (*N* = 1,080) of Saudi women from various age categories, different social, educational, and employment status, and geographical regions. Pooled confirmatory factor analysis was using to determine the reliability and validity of the scale. As a result, the reliability and validity of the entire model were satisfied. Specifically, the composite reliability values fell in the range between 0.804 and 0.883. Meanwhile, the convergent validity was achieved as these values produced from each construct were higher than the acceptable limit of 0.50. Also, the construct validity of fitness indexes (e.g., comparative fit index, incremental fit index, Tucker–Lewis Index, root mean square error of approximation, and chi-square over degree of freedom) was achieved. In its final form, multicultural psychological empowerment scale for Saudi Women included (26) phrases distributed over its four dimensions. The study suggested systematic measurements of psychological empowerment for Saudi women from different categories, targeted with therapeutic, preventative and developmental visions, plans, and programs to determine the extent of their psychological empowerment.

## Introduction

The empowerment of women is an integral part of human rights and development. Development as a whole is responsible for decreasing inequality between men and women, while the empowerment of women can speed up the process of development (Batool et al., 2016). The empowerment of women is a multidimensional process, which occurs within sociological, psychological, and economic contexts at various levels, i.e., individual, group, and community levels (Zimmerman, 1995; Malhotra et al., 2002; Thani and Mokhtarian, 2012; Manuere and Phiri, 2018). Women empowerment is not necessarily about giving them power, it is enabling them to exercise that power. Many women already have plenty of power in their wealth of knowledge and motivation, but they are not always a member of an empowered class (Mishra, 2014; Valarmathi and Hepsipa, 2014).

The empowerment of women is “the processes through which they know and then correct the gender issues that impede their development” (Longwe, 1998). Empowering women is a prerequisite for creating a good nation, when women are empowered, a society with stability is assured. Empowerment of women is essential as their value systems lead to the development of a good family, good society and ultimately a good nation (Gupta, 2018). Women empowerment is defined as “the process through which women acquire the ability to make strategic life choices in a context where this ability was previously denied to them” (Huis et al., 2017). According to Lazo (1995), empowerment gives a woman a new perspective on what is negative about her current situation and enables her to see what is within her reach to attain a better situation for herself (Lazo, 1995). Huis et al. (2017) have proposed a three-dimensional model of women empowerment, which posits that the empowerment of women can take place at three distinct levels, namely, (1) the micro-level or personal dimension manifested in their self-efficacy, (2) the meso-level or relational dimension manifested in the bargaining power that women possess and social capital, and (3) the macro-level or societal dimension manifested in gender disparity in human development.

### Women Empowerment in Saudi Arabia

Recent decades have seen governments across the world committing themselves more resolutely to the empowerment of women. The empowerment of Saudi women economically, socially, and politically has become a key priority since it occupies an advanced position among the concerns of a modern developmental thought (Al-Tarik, 2014, pp. 11, 12). Women empowerment is a policy urgently needed in Saudi Arabia where gender difference is still widely observed. The Global Gender Gap Index for Saudi Arabia was 0.524 in 2006, 0.6059 in 2014, and 0.599 in 2020 (Global Gender Gap, Report, 2020). So, Saudi Arabia has launched some decisions regarding the empowerment of Saudi women:

(1) Election of women into the local chambers of commerce, 2005.
(2) Thirty women were appointed to the Consultative Assembly of Saudi Arabia 2013.
(3) The participation of women as voters and candidates for the first time in municipal councils in 2015.
(4) Issuing driving licenses for women in 2018.
(5) Thirteen women were appointed to the Council of the Human Rights Commission.
(6) Granting 244 women a license to practice the legal profession.
(7) The issuance of a set of amendments to the Travel and Civil Status System 2019, including a woman obtaining a passport just like a man, the right to travel after reaching the age of 21 years.
(8) The issuance of a set of decisions on judicial dealings in 2019, including approval of the fund to spend on the children of women during litigation periods related to marital disputes and criminalizing forcing women to marry under coercion.
(9) The vision of the Kingdom of Saudi Arabia 2030, launched in 2016, included a concern for women, which was evident in increasing the percentage of participation of women in the labor market from 22 to 30% and increasing women in top jobs “grade 11 and above” from 1.5 to 5%.

Empowering Saudi women were tackling in social studies: Al-Qablan (2012) pointed out that woman are considered a dynamic and vital element in society if they have the opportunity to develop their capabilities and skills. Al-Meezar (2017) showed that Islamic religion has raised the status of women, made them equal to men, and given them their rights, and the Noble Quranic texts address men and women equally. Bin Shalhoub (2017) pointed out that the most prominent features of empowering Saudi women include providing women with their legitimate rights in society and enhancing the personal and social strength of women. Hassan (2015) showed the progress of women empowerment in the present time as compared with the past with an emphasis on the need to change the fruitless legacies that attempt to isolate women from society. Al-Fayez (2011), Abu Khedir (2012), Al-Abdulkarim (2014), Al-Khmashi (2014), and Al-Kasr (2015) showed many obstacles that have led to a decrease in the effectiveness of the role of a Saudi woman such as ignorance of their rights, lack of awareness of their developmental role, the lack of appreciation of the community for their developmental role, and societal discrimination between the sexes.

There is an important aspect that must be pointed out here: “cultural context of empowerment of Saudi women.” The subordinate position of women is not natural but socially constructed and forced upon them. Elimination of the cultural devaluation of Saudi women is required so they are seen as people and not as the constructed feminine identity of the subservient (Rao, 2017; Selamu and Singhe, 2017). The long-lived patriarchal structures in Saudi Arabia have become readily accepted. Culturally imposed rules and regulations are deep-rooted and dominant, while experimentally acquired precedents have defined responsibility and status of women in the society as one of subordination. As a result, the subordination of women is an unconscious process. Women do not realize that they play willingly into the hands of patriarchal rule and powers: “power is gendered.” Most significant is the failure to actively engage men in gender equality work (United Nations Development Programme [UNDP], 2015; Huis et al., 2017). Indeed, women empowerment can only be developed through interaction between the women and the cultural context (Mosedale, 2005; Narayan, 2005; Haase, 2011; Kurtis and Adams, 2015; Dutt et al., 2016).

### Psychological Empowerment of Women

A good mental health is a fundamental part of women well-being, so the empowerment of women cannot be complete without their psychological empowerment (PE). There is a dearth of programs addressing mental health issues to secure PE for women (Yadav, 2019). It is generally agreed that empowerment is a process developed from the bottom-up and is not something that can be framed as a top-down strategy. Henceforth, any strategy focused on empowerment must support women to analytically evaluate their own situation and outline the necessary alterations within society (Batool et al., 2016). Most importantly, from a psychological perspective, strategies must build upon the “psychological capital” of women. These approaches recognize that the vitality and resilience of women are “protective factors” that should be nurtured (Spreitzer and Doneson, 2005; Smalley et al., 2010).

The importance of PE is highlighted by Oladipo (2009). Oladipo showed that although there have been various economic and communal programs of empowerment delivered, they have proven unsuccessful. This could be due to the fact that those for whom the programs were intended lacked the necessary psychological make-up. Although empowerment initiatives by feminist movements, the state, and non-governmental organizations may create conditions favoring empowerment, women still may not feel empowered. Therefore, there is a distinction between creating conditions conducive for empowerment and the actual realization of empowerment (Spreitzer, 1995). PE is understood to be “an individual’s cognitive state which is characterized by their sense of perceived control, competence, and personal aspirations” (Oladipo, 2009).

With regard to women empowerment, PE has the effect of increasing the self-confidence of a woman, strengthens her bargaining power, expands her freedom of choice, and develops her coping capacities within the home (Parveen and Leonhauser, 2005). The four indicators of PE of women are impact, significance, competence/self-efficacy, and choice/self-determination (Hackman and Oldham, 1980; Ashforth, 1989; Bell and Staw, 1989; Brief and Nord, 1990; Gist and Mitchell, 1992; Batool and Batool, 2018).

Spreitzer (1995) suggested that several assumptions can be made when defining PE. First, empowerment is not a permanent personality trait applicable in all situations. Second, empowerment can be an ongoing variable; rather than being empowered or not, people may feel they possess varying levels of empowerment. Third, empowerment is not a global concept that can be applied to different life situations and roles but is an idea relevant to specific activities. Empowerment takes on different meanings and forms in different situations and can vary from person to person or for the same person at different life stages. The components of PE are not gained in any particular order, and one does not necessarily lead to another; each component can be found in varying degrees in the same individual (Zimmerman, 1995, 2000). Pandey (2016) highlighted how self-efficacy and perceived resource adequacy are important for the transformation of initiatives of structural empowerment to PE. There are three steps for PE of women (Yadav, 2019), namely, gaining a sense of self-determination and personal efficacy or competency, reducing isolation and having opportunities to develop a sense of belonging, and involvement in social and economic decision-making at all levels.

Psychological empowerment is a formative latent constructs. Spreitzer (1995), Zimmerman (1995), Seibert et al. (2011), Christens (2012), Peterson (2014), and Miguel et al. (2015) provided evidence that supports the formative measurement model of PE. Later, Thomas and Velthouse (1990) defined PE as the presence of four cognitive assessments, namely, impact, meaningfulness, competence/self-efficacy, and choice/self-determination; psychological studies emphasized PE as a combination of these four cognitive components.

## Literature Review of Psychological Empowerment

Since the 1980s, many studies about PE have been conducted in the workplace (Bell and Staw, 1989; Thomas and Velthouse, 1990; Spreitzer, 1995; Stander and Rothmann, 2010; Lee and Wei, 2011; Thani and Mokhtarian, 2012; Quiñones et al., 2013; Zhang et al., 2014; Miguel et al., 2015; Mishra, 2016; Meng and Sun, 2019; Karimiha, 2020). In Saudi Arabia, there had been also an interest in PE in the workplace (Al-Jardi, 2012; Radwan, 2015; Abu Zaid, 2016; Halim, 2017; Al-Mahamid and Kasasbeh, 2018). The previous studies found that PE can effectively promote job performance, job satisfaction, organizational commitment, work engagement, social capital, leadership competence, organizational creativity, and participation behaviors. So, it can be concluded that PE, within the range of studies mentioned, is linked to organizational and institutional work, which undoubtedly concerns an important aspect of the life-work of a woman, and none of these topics pertains specifically to women. Seemingly, there is only one study that worked on finding out the determinants of PE of women. A research was carried out by Batool and Batool (2017) to construct a measure of global PE for women (Batool et al., 2016). Global Psychological Empowerment Scale has five dimensions (i.e., meaningfulness, competence/self-efficacy, choice/self-determination, impact, and problem-focused coping), and a sample of women was recruited from five major cities of Pakistan.

## Why Psychological Empowerment for Saudi Women Rational?

Attention to the PE of Saudi women in the current article was based on the following considerations; women are psychological entities and this must be taken into account during the formulation of policies affecting them, otherwise, various economic and social empowerment programs may fail to achieve the expected results; when women are psychologically empowered, there will be a change in their attitude, cognition, and behavior; women must empower themselves because empowerment is essentially a bottom-up rather than a top-down process; the sociocultural and economic status of Saudi women remains low due to the prevailing patriarchal, feudal systems of a country; and PE is an important intermediate stage to achieving the various dimensions, areas, and levels of empowerment. So, achieving empowerment for women will take more than changes in law, strategies, or policies to change unfair practices.

## Objectives

Based on the above discussion, the research objective of this study was to construct the Multicultural Psychological Empowerment Scale for Saudi Women (MPES–SW), depending on the four dimensions of psychological empowerment within the scale to appraise psychological empowerment (i.e., meaningfulness, competence/self-efficacy, choice/self-determination, and impact). Based on these main considerations:

(A) The women empowerment was measured using different indicators. The Gender Development Index is a measurement for gender inequalities in the three dimensions covered by the Human Development Index, namely, life expectancy, education, and income. Another tool used is the Gender Empowerment Measure, which seeks to measure relative female representation in economic and political power. It considers gender gaps in political representation, in professional and management positions in the economy, as well as in incomes (Selamu and Singhe, 2017). In addition, there are limited number of scales that measure female empowerment, such as Women’s Empowerment Scale (Schuler et al., 1997), Women Empowerment Scale (Al-Qahtani et al., 2018), Healthcare Empowerment Questionnaire (Mohebbi et al., 2018), and Women’s Economic Empowerment (Laszlo and Grantham, 2017).
(B) The interest in PE has guided researchers toward the development of several scales intended to measure levels of PE exclusively in a workplace setting (except the last scale); nomological network of empowerment (Thomas and Velthouse, 1990), including PE scale to quantify the degree to which an individual is psychologically empowered at the workplace (Spreitzer, 1995); leader empowering behavior (Konczak et al., 2000), Scale of Psychological Empowerment among school teachers (Wang and Zhang, 2012), and Global Psychological Empowerment Scale for Women (Batool and Batool, 2017).
(C) So far, there is only one measure of PE of women, i.e., Global Psychological Empowerment Scale for Women (Batool and Batool, 2017). It was applied in Pakistani society, which was completely different from the Saudi society. To the best of the knowledge of authors, there is no valid and reliable scale of PE available for Arabian women, especially Saudi women, in light of the culture of Saudi society, the status of women in it, and the development that has befallen society in the areas of empowering women.

## Theoretical Framework: Power of Empowerment Perspective

The simple idea of PEP of the author is based on the premise that women gaining power, or describing a woman as powerful, is a simultaneous indication of being a “psychologically empowered woman.” Therefore, the concepts of power and PE are viewed as follows;

(I) Psychological empowerment is a process and an outcome. The process includes providing women with meaning, competence, influence, and self-determination. The result is an empowered woman who has practiced the experience of empowerment and possessed or was present in an empowered environment, since the practice of empowerment, as meaning, competence, influence, and self-determination, is the result of the empowerment process.
(II) Psychological empowerment does not give women power. Rather, it allows and helps them to wield that power and expand their practice in various areas of life. Hence, empowerment reflects power, and therefore, there is no empowerment without power nor power without empowerment.
(III) Power and empowerment concepts are simultaneously inseparable, possessed, and practiced.
(IV) No matter how organizations, communities, and nations establish favorable conditions for empowerment, if women have not actively engaged in the experience of actual empowerment, then empowerment initiatives, policies, and programs will not have the desired effect.
(V) A “strong empowered woman” is a woman who feels she has meaning, competence, influence, and self-determination. She is, in other words, “a psychologically empowered woman.”

## Methodology

### Scale Development

#### Determined the Goals of the Scale

Main goal: measure the extent of PE of Saudi women in multiple cultures. Sub-goals were measuring indicators of meaningfulness, impact, self-efficacy, and self-determination of Saudi women.

#### Sub-scale Dimensions Were Determined and Defined the Concepts of the Scale Procedurally

Psychological empowerment is “a cognitive state and an increasing inner feeling of meaningfulness, impact, self-efficacy, and self-determination of an woman in different contexts, times, and life domains to the height of their potential, abilities, and power.” Meaningfulness is “the value and importance a woman gives to her roles, duties, work, and purpose in life.” Impact is “the degree to which a woman feels that she can exert strategic influence on her family, and social circle, and perseverance in difficult situations.” Self-efficacy is “the degree to which a woman feels that she is able to perform different tasks with skill and display confidence in the ability to exercise control over her own behavior and social environment.” Self-determination is “the sense of autonomy in taking initiative, making decisions, and demonstrating the degree of autonomy in work, relationships, behaviors, and processes.”

#### (Fifty Six) of the Items of the Initial Scale

(Fifty Six) of the items of the initial scale were developed from; literature and previous scales about PE of women; interviews with (5) focus groups formed from different categories of Saudi women; and interviews of a stakeholder from feminist movements, the state policy makers, and non-governmental organizations.

#### A 5-Point Likert Format Was Assigned

1 = Strongly disagree, 2 = disagree, 3 = indecisive, 4 = agree, and 5 = strongly agree.

#### Face Validity

The MPES-SW was applied in its initial form to a limited sample of Saudi women, consisting of (257) women from different age groups and statuses (i.e., social, economic, educational, and administrative) and geographical regions. The MPES-SW items were restructured based on opinions of participants about the characteristics of clarity and simplicity of the phrasing. The first restructuring resulted in retaining 48 ostensibly valid phrases and deleting (8) invalid phrases.

#### Content Validity

Multicultural psychological empowerment scale for Saudi Women dimensions and items were presented to four judges in social psychology and gender. For the second time, MPES-SW items were restructured based on the consensus three of the judges (75%), resulted in retaining (40) phrases that were theoretically and structurally valid and deleting (8) phrases that were not valid.

### Target Population and Sampling Techniques

Multicultural Psychological Empowerment Scale for Saudi Women applied to (*N* = 1,080) Saudi women. The electronic link of MPES-SW was presented to the target community due to the wide range of administrative geographical regions, the multiplicity of characteristics of the respondents, and the use of technology by the majority of Saudis.

There was diversity in the characteristics of the study sample, as Saudi women from different cultures were represented. Table 1 shows the characteristics of the study sample, where the age distribution was as follows: 40.5% in the age category 21–30 years, 39.5% in the age category 31–40 years old, 13.5% in the age category 41–50 years, and 6.5% in the age category 51–60 years old. In educational status, the distribution was 67.2% had a university education, 19.2% had preuniversity education, and 13.6% had postgraduate education. In marital status, the percentage of married women was 45.1%, with 29.4% single, 17.5% divorced, and 8.0% widowed. In employment status, 56.3% of women were employed, and 43.7% were housewives. Finally, the distribution of respondents according to geographical region was as follows: 37.6% from the middle region (Riyadh), 24.8% from the west region (Jeddah), 14.3% from the east region (Dammam), 13.1% from the north region (Ha’il), and 10.2% from south region (Najran).

**TABLE 1:** Characterization of the sample (*N* = 1,080).

Variables	Categories					
Age	21–30	31–40	41–50	51–60	Total	
*N*	437	427	146	70	1,080	
%	40.5	39.5	13.5	6.5	100%	

**Educational status**	**Preuniversity**	**University**	**Postgraduate**	**Total**		

*N*	207	726	147	1,080		
%	19.2	67.2	13.6	100%		

**Marital status**	**Single**	**Married**	**Widowed**	**Divorced**	**Total**	

*N*	318	487	86	189	1,080	
%	29.4	45.1	8.0	17.5	100%	

**Employment status**	**Employee**	**Housewife**	**Total**			

*N*	608	472	1,080			
%	56.3	43.7	100%			

**Geographical regions**	**North Ha’il**	**South Najran**	**Middle Riyadh**	**East Dammam**	**West Jeddah**	**Total**

*N*	142	110	406	154	268	1,080
%	13.1	10.2	37.6	14.3	24.8	100%

## Results

As a preliminary study, a pilot test is important for any empirical research and usually recommended by applied researchers to address a variety of issues through two statistical methods such as Exploratory Factor Analysis (EFA) and Cronbach’s alpha. From here, the number of components can be identified and confirmed by the reliability approach. As a result, the instrument is modified using the EFA suggestions before administering the questionnaire for field work purpose.

### Factor Analysis Results

#### Exploratory Factor Analysis

The factor analysis is widely used in the variety of research fields such as management, education, psychology, finance, accounting, and so forth. It is always considered as a method of choice in exploratory research and pilot testing when interpreting the structured survey whereby also suitable for the present study. Using EFA, the researchers have no sufficient information of the number of the factor proposed in the study (Williams et al., 2010). Thus, it allows the researchers to explore the number of dimensions or components to generate a new model and then will be used for further examinations using other relevant statistical methods. The dimension or components are relatively called as a latent variable assessed by a set of items (Pett et al., 2003; Henson and Roberts, 2006; Afthanorhan et al., 2020). The EFA method is aimed to reduce the large number of variables to a smaller set of variables, which clustered in the respective variables (Dobni, 2008; Aimran et al., 2017; Afthanorhan et al., 2019; Majid et al., 2019). As suggested by previous scholars, suitability of data must be considered at the initial phase of inferential statistics before moving to any statistical analysis. Thus, we do the EFA method for the pilot testing, which involves 108 respondents. The sample size might be arguably small for the present study, but this method actually only used for the pilot testing. According to Hair et al. (2017) and Afthanorhan and Aimran (2020), the acceptable sample size is at least 100 when using the EFA method.

In the EFA method, the Kaiser Meyer Olkins (KMO) test assesses the sampling adequacy, while the Bartlett’s test of sphericity measures the suitability of data at hand for the factor analysis. The factor analysis is indicated valid if the value of KMO index is higher than 0.60 and Bartlett test is significant at 0.05 (Hair et al., 2017; Afthanorhan and Aimran, 2020). Therefore, the data are considered adequate and sufficient for the current study. Table 2 presents the result of the test. For designing the factor, the study must extract factors to analyze the total variance accounted from these data and generate the value of factor loading for each variable applied in the study. The principle component analysis (PCA) is the most commonly used for extraction method. The PCA has several advantages compared with the other extraction method as it does not need stringent assumptions and is efficient for the process that takes place in smaller number of components or dimensions. As a result, the PCA is often being the default method in much of the statistical software. Moreover, the PCA is also suggested when the study has no priori information or lack of evidence (Gorsuch, 1983). In the meantime, the rotation method is an approach to determine the number of factors that are produced from the data. There are several available approaches for the rotation method such as Varimax, Direct Oblimin, Quartimax, Equamax, and Promax. Among these approaches, Varimax rotation is the most commonly used in EFA developed by Thompson (2004). It is recommended because it will capture maximum of variances yielded from the data for further assessment on determining the set of variables underlying on each factor. Nonetheless, the goal of this research is to provide easier interpretation of results. Thus, to construct 40 items, the study performed PCA with Varimax rotation to test the validity of the factors. The EFA results are shown in Tables 3, 4. Specifically, the KMO value is 0.782, which meets the acceptable threshold as shown in Table 2; later, the PCA with Varimax rotation is performed simultaneously. It was observed that four components were yielded with 57.627% of cumulative variance as depicted in Table 3; the factor loading results are shown in Table 4. It shows that 6 (i.e., Item 8, Item 21, Item 26, Item 33, Item 36, and Item 37) out of 40 items have a poor loading that is lower than 0.50. Therefore, these items were dropped from further analyses as it were expected not worth for measuring the factor. So factors 1, 2, 3, and 4 have 9, 10, 8, and 7 items, respectively. Each factor also yielded a reliability coefficient of alpha greater than 0.70 as recommended by Nunnally and Bernstein (1994) as presented in Table 5.

**TABLE 2:** Kaiser Meyer Olkins (KMO) results (KMO and Bartlett’s test).

Kaiser-Meyer-Olkin measure of sampling adequacy		0.782
Bartlett’s test of sphericity	Approx. Chi-Square	3,509.510
	df	780
	Sig.	0.000

**TABLE 3:** Total variance explained.

Items	Initial eigenvalues			Extraction sums of squared loadings			Rotation sums of squared loadings		
	Total	% of Variance	Cumulative%	Total	% of variance	Cumulative%	Total	% of variance	Cumulative%
1	15.987	39.966	39.966	15.987	39.966	39.966	6.441	16.102	16.102
2	2.924	7.310	47.276	2.924	7.310	47.276	6.433	16.083	32.185
3	2.245	5.614	52.890	2.245	5.614	52.890	5.095	12.737	44.922
4	1.895	4.737	57.627	1.895	4.737	57.627	5.082	12.705	57.627
5	1.586	3.965	61.592						
6	1.302	3.256	64.847						
7	1.205	3.013	67.861						
8	1.144	2.860	70.721						
9	1.044	2.610	73.331						
10	1.000	2.500	75.831						
11	0.870	2.176	78.007						
12	0.819	2.048	80.055						
13	0.758	1.895	81.950						
14	0.718	1.796	83.745						
15	0.657	1.642	85.387						
16	0.573	1.431	86.819						
17	0.474	1.186	88.005						
18	0.453	1.132	89.137						
19	0.416	1.040	90.177						
20	0.380	0.951	91.128						
21	0.373	0.933	92.061						
22	0.358	0.895	92.956						
23	0.321	0.804	93.759						
24	0.282	0.706	94.465						
25	0.266	0.665	95.131						
26	0.238	0.596	95.727						
27	0.220	0.551	96.278						
28	0.195	0.488	96.766						
29	0.181	0.452	97.217						
30	0.172	0.431	97.648						
31	0.163	0.408	98.057						
32	0.151	0.377	98.434						
33	0.133	0.331	98.765						
34	0.109	0.274	99.039						
35	0.101	0.252	99.290						
36	0.084	0.210	99.500						
37	0.076	0.189	99.690						
38	0.060	0.149	99.839						
39	0.043	0.108	99.946						
40	0.021	0.054	100.000						

*Extraction method: principal component analysis.*

**TABLE 4:** Rotated component matrix^a^.

Items	Component			
	1	2	3	4
ITEM 1				0.512
ITEM 2				0.542
ITEM 3				0.839
ITEM 4				0.557
ITEM 5				0.618
ITEM 6				0.651
ITEM 7			0.621	
ITEM 8				
ITEM 9	0.509			
ITEM 10	0.573			
ITEM 11		0.722		
ITEM 12		0.511		
ITEM 13		0.771		
ITEM 14		0.655		
ITEM 15		0.762		
ITEM 16		0.579		
ITEM 17				0.544
ITEM 18				0.692
ITEM 19	0.504			0.546
ITEM 20		0.535		
ITEM 21				
ITEM 22	0.799			
ITEM 23	0.771			
ITEM 24	0.808			
ITEM 25	0.663			
ITEM 26				
ITEM 27	0.586			
ITEM 28	0.784			
ITEM 29	0.755			
ITEM 30		0.523		
ITEM 31		0.535		
ITEM 32		0.569		
ITEM 33				
ITEM 34			0.703	
ITEM 35			0.657	
ITEM 36				
ITEM 37				
ITEM 38			0.676	
ITEM 39		0.513		
ITEM 40			0.555	

*Extraction method: principal component analysis.*

*Rotation method: varimax with Kaiser normalization.*

*^a^Rotation converged in 16 iterations.*

**TABLE 5:** Reliability result.

Factor	1 Meaningfulness	2 Impact	3 Self-efficacy	4 Self determination
Total items	9	10	8	7
Cronbach’s alpha	0.916	0.910	0.826	0.897

#### Confirmatory Factor Analysis

The next step in our evaluating of PE construct was to perform confirmatory factor analysis (CFA), as continuous distribution for each construct was expected. For the purposes of CFA, a sample size of 1,080 for this study may be considered large and adequate for further investigations. The Analysis Moment of Structure (AMOS 21.0) software is used to perform the CFA. In the AMOS software, the maximum likelihood estimator has been used to generate the parameter estimates for each construct and path. In addition, the maximum likelihood estimator was recognized as the best linear unbiased estimator (Byrne, 2013; Westland, 2015; Afthanorhan et al., 2020), which is particularly useful for the present study.

The latent construct in the current study included meaningfulness, impact, self-efficacy, and self-determination. These latent constructs were the subscale of the PE. This type of classification has been recommended from EFA during the pilot stage. Thus, meaningfulness, impact, self-efficacy, and self-determination factors had 9, 10, 8, and 7 indicators, respectively, for a total of 34 indicators.

As suggested for this type of evaluation, a pooled CFA allowed all latent constructs to be examined comprehensively in terms of their correlations, factor loading, and fitness. It is a necessary approach when performing the structural equation modeling (SEM) method to reduce the risk of validity. Moreover, the maximum likelihood method of the covariance structure analysis was used to generate the outcome for each latent construct. To examine the overall model fit, the parsimonious fit [i.e., chi-square over degree of freedom (Chisq/df)], incremental fit [i.e., comparative fit index (CFI), incremental fit index (IFI), and Tucker–Lewis Index (TLI)], and absolute fit [i.e., root mean square error of approximation (RMSEA)] were used. The model achieved satisfactory results when the value of each fitness index met the acceptable limit as presented in Table 6.

**TABLE 6:** Indicators of good fit of the model to the data (*N* = 1,080).

Indicators of good fit	Chisq/df	CFI	IFI	TLI	RMSEA
	(<5.0)	(>0.9)	(>0.9)	(>0.9)	(<0.08)
Value	4.905	0.908	0.908	0.884	0.08

*Chisq/df, chi-square over degree of freedom; CFI, comparative fit index; TLI, Tucker–Lewis Index; IFI, incremental fit index; RMSEA, root mean square error of approximation.*

**FIGURE 1:**
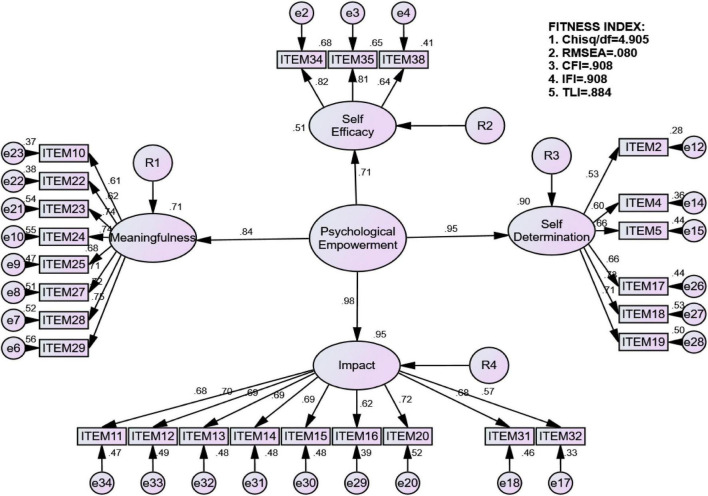
Exploratory construction of Multicultural Psychological Empowerment Scale for Saudi Women.

The original measurement model using 34 indicators failed to meet the acceptable limit as indicated by all fitness indexes of RMSEA > 0.08; all incremental fit < 0.90; and Chisq/df > 5.0, suggesting the need for modification. It was observed that some indicators did not load on the respective construct, whose value of factor loading was lower than 0.60. According to the studies by Hair et al. (2017) and Afthanorhan et al. (2020), the indicator is perfectly loaded in the model when the value of an indicator loading is higher than 0.60, which can enhance the construct reliability. Following these recommendations, 8 indicators were dropped (i.e., Item 9, Item 40, Item 39, Item 1, Item 3, Item 6, Item 7, and Item 30) from further assessment. Thus, the revised measurement model using 26 indicators, namely, “4 for meaningfulness, 10 for impact, 7 for self-efficacy, and 5 for self-determination” (Figure 1) reproduce adequately the covariance matrix as indicated by RMSEA < 0.08; CFI and IFI > 0.90; and Chisq/df < 5.0 values. In addition, all factor loadings were above 0.60 indicating construct validity. Next, the convergent validity and reliability indicated by average variance extracted (AVE) and composite reliability (CR) were determined. The value of CR for each latent construct was between 0.804 and 0.883, indicating that all construct reliabilities were satisfied. Other than CR, the results showed that all latent constructs had AVE values of more than 0.50, and the values of AVE were in the range of 0.504 and 0.581.

### Criterion Validity Results

The value of the correlation coefficient was (0.788^**^) between the sample degrees in MPES-SW and World Health Organization Quality of Life-BREF, which is statistically significant at the level of 0.01.

### Internal Consistency Results

Table 7 shows that the correlation coefficients between the degree of each item, the degree of the dimension to which it belong, and the total degree of the scale is statistically significant at the level of significance (0.01). This means that the items of the scale are consistent with the dimension to which it belongs and with the total degree of the scale. Table 8 shows that the correlation coefficients between the dimensions of MPES-SW and the total degree of the scale are statistically significant at the level of significance (0.01). This means that the dimensions of the Psychological Empowerment Scale are consistent with the scale.

**TABLE 7:** Correlation coefficients between the degree of each item and the degree of the dimension to which it belongs and the total score of the scale.

Items	Correlation coefficient with		Correlation coefficient with		Correlation coefficient with		Correlation coefficient with	
	Dimension	Total degree	Dimension	Total degree	Dimension	Total degree	Dimension	Total degree
	Meaningfulness		Impact		Self-efficacy		Self-determination	
1	0.703**	0.552**	0.746**	0.607**	0.694**	0.678**	0.709**	0.719**
2	0.712**	0.546**	0.714**	0.671**	0.676**	0.547**	0.772**	0.647**
3	0.679**	0.532**	0.746**	0.622**	0.739**	0.670**	0.761**	0.650**
4	0.733**	0.594**	0.718**	0.672**	0.776**	0.663**	0.760**	0.625**
5	0.728**	0.646**	0.752**	0.625**	0.726**	0.656**	0.784**	0.644**
6	0.692**	0.553**	0.703**	0.581**	0.689**	0.619**	0.630**	0.527**
7	0.734**	0.618**	0.694**	0.624**	0.750**	0.676**	0.665**	0.551**
8	0.713**	0.578**	0.723**	0.679**	0.782**	0.642**	0.716**	0.604**
9	0.572**	0.498**	0.734**	0.651**	0.764**	0.718**	0.645**	0.571**
10	0.646**	0.626**	0.769**	0.668**	0.638**	0.632**	0.538**	0.445**

**: p-value < 0.05.

**TABLE 8:** Correlation coefficients between the dimensions of the Psychological Empowerment Scale and the total degree of the scale.

Variables	Correlation coefficient with overall degree
Meaningfulness	0.835**
Impact	0.875**
Self-efficacy	0.895**
Self-determination	0.856**
Total degrees	0.606**

**: p-value < 0.05.

## Discussion

Since the recognition of PE according to the conclusion of Oladipo (2009), there have not been valid and reliable scales to measure the PE of Arab women in general and Saudi women in particular. So, MPES-SW was developed by four indicators, namely, impact, meaningfulness, competence/self-efficacy, and choice/self-determination.

Initially, the EFA method was performed using the pilot data to determine the number of components yielded from data at hand besides examining the value of factor loadings. From these results, six items were removed from further examination due to poor factor loadings (lower than 0.60). Moreover, four dimensions with items also were yielded from the EFA. Subsequently, the field work is performed using a new version of questionnaire (after removing six items from an original questionnaire) on 1,080 samples. To confirm the validity of the measurement model, the pooled CFA was performed with the AMOS software. Eight indicators were dropped, Thus, the revised measurement model contained 26 indicators, namely, “4 for meaningfulness, 10 for impact, 7 for self-efficacy, and 5 for self-determination.” MPES-SW also had criterion validity of 0.788^**^ and internal correlation coefficient of total degree (0.606^**^). Using this method, all the reliabilities (i.e., CR) and validities (i.e., construct validity, convergent validity, and discriminant validity) were satisfied, and we concluded that the proposed model is reasonable for the future research.

Descriptively, the first dimension of MPES-SW included indicators of how Saudi women feel regarding meaningfulness, the value of her duties, the impact of achieving her goals on various aspects of her life, the benefit of her community participation, and opportunities to develop her personality. The second dimension included indicators of how Saudi women feel regarding impact, her influence on everyone around her, her ability to help all members of her social circle, her ability to persuade others in various situations, creating a positive and moral atmosphere in all around her, passing her thoughts to others, advocacy of the rights of others, having clear positions on various issues of life, making a positive moral difference in her various relationships, giving power to those who need it from her social circle, and establishing effective alliances with others. The third dimension included indicators of how Saudi women feel regarding self-efficacy, controlling her behavior in the situations that need it, investing the empowerment opportunities in various fields, dealing effectively with new situations, modifying her unwanted life statuses, having good problem-solving skills, managing the various types of available time, and gaining everything useful that contributes to achieving her goals. The fourth dimension included indicators of how Saudi women feel regarding self-determination, taking the initiative in various life situations, making her decisions with complete independence, instituting her relationships freely, practicing freedom of choice in situations that need it, acting freely on all her matters, and taking responsibility for her decisions.

The existence of a scale with validity and reliability to measure the PE of women in the Saudi society, “having deep-rooted and dominant culturally imposed meanings,” can contribute greatly to identifying the extent to which Saudi women feel regarding social, educational, economic and political empowerment processes and whether they are on a path of empowerment. So, Saudi women can correct gender issues that impede their development and acquire the ability to make strategic life choices in a context where this ability was previously denied to them. Therefore, Saudi women can feel positively about empowerment under favorable conditions to empowerment through programs, policies, and visions of the state. The psychological perspective of empowerment recognizes that the vitality and resilience of women are “protective factors” that should be nurtured as buffers (Spreitzer and Doneson, 2005; Smalley et al., 2010). Returning to the simple idea of empowerment by the author, we concluded that “a strong empowered woman” is a woman who feels meaning, competence, influence, and self-determination, making her a “psychologically empowered woman.”

## Multicultural Psychological Empowerment Scale for Saudi Women Scores and Considerations

### Multicultural Psychological Empowerment Scale for Saudi Women Scores

Multicultural Psychological Empowerment Scale for Saudi Women scores were determined as (total maximum score = 130, total middle score = 78, total minimum score = 26). Therefore, the PE of Saudi women is measured in a range of 26°–130°.

### Considerations of (Multicultural Psychological Empowerment Scale for Saudi Women) Application

(a) Merging (MPES-SW) phrases with each other without sub-dimension headings, so that responses do not come in one direction with a key of MPES-SW.
(b) Determining the meaning of the score of a woman on MPES-SW, by converting her raw score into a standard score.

## Conclusion

Multicultural Psychological Empowerment Scale for Saudi Women is a valid and reliable scale in Saudi Arabia from a multicultural point of view. Designing a scale within a society such as Saudi Arabia, which has “a deep-rooted and dominant culturally imposed view of women,” is commendable, especially when considering a large sample size of Saudi women from various cultures. Perhaps this measure is the beginning of Saudi women becoming aware of what is inadmissible about her situation, perceiving a better situation and understanding how she can improve it with support from relevant institutions interested in empowering Saudi women. In light of this, the three dimensions of empowerment of Saudi women can be categorized into at least three distinct levels, namely, the micro-level or personal dimension, the meso-level or relational dimension, and the macro-level or societal dimension. As a result, possible applications of MPES-SW are as follows;

(a) Systematic measurements of different groups of Saudi women targeted with therapeutic, preventive, and developmental visions, plans, and programs, to determine the extent of their PE, as an important and priority dimension of their empowerment.
(b) Measuring the PE of Saudi women as one of the dimensions of their mental health.
(c) Measuring the PE of Saudi women throughout the various stages of her life, especially the critical periods of strength and weakness.
(d) Measuring the PE of Saudi women in a society during its initial steps toward changing its cultural context toward women.

## Limitations

The status of Saudi women is linked to a traditional conservative social structure, which is linked to the phenomenon of the dominance of the masculine/masculine culture, which is the result of the interaction of a number of cultural, social, economic, and political factors, as culturally imposed meanings are deep-rooted. Despite the good steps taken by the Saudi government to empower women, the Global Gender Gap Index reveals a discouraging picture of women empowerment in Saudi Arabia. The Global Gender Gap Index for Saudi Arabia is 2006 (0.524), 2014 (0.6059), and 2020 (0.599), noting that the highest possible score is 1 (equality or better for women). Empowerment of Saudi women is still relatively complex, given the geographical and cultural differences in the Kingdom and also due to the different impact of state policies to empower Saudi women on different groups of women, and then there is a difference between creating favorable conditions for empowerment and the experience of empowerment. Therefore, MPES-SW faces a wide and complex cultural, geographical, and development range—even with its application across multiple cultures—which makes sense of PE of women different across cultures, regions, and times. Therefore, MPES-SW needs future studies with different categories of Saudi women across different situations and times (it may be a new policy for women such as allowing them to drive for the first time, or allowing them to run for office and vote for the first time in municipal councils, or any future event in this regard). Because the Kingdom is going through a period of change, that coincides with new policies regarding women.

## Data Availability Statement

The original contributions presented in the study are included in the article/Supplementary Material, further inquiries can be directed to the corresponding author.

## Ethics Statement

The studies involving human participants were reviewed and approved by Research Ethics Committee (REC), Ha’il University, Saudi Arabia, 15-3-2021, University president letter number 42-5-38862, 02-08-1442 H. Written informed consent for participation was not required for this study in accordance with the national legislation and the institutional requirements.

## Author Contributions

HM: abstract, introduction, theoretical and conceptual framework, objectives, methodology, results, discussion, and conclusion. AA: statistical analysis. EA: participated in data collection, Arabic studies translation, and logistics. All authors contributed to the article and approved the submitted version.

## Conflict of Interest

The authors declare that the research was conducted in the absence of any commercial or financial relationships that could be construed as a potential conflict of interest.

## Publisher’s Note

All claims expressed in this article are solely those of the authors and do not necessarily represent those of their affiliated organizations, or those of the publisher, the editors and the reviewers. Any product that may be evaluated in this article, or claim that may be made by its manufacturer, is not guaranteed or endorsed by the publisher.
