# Supplementary material for: Multicultural Psychological Empowerment Scale for Saudi Women
Source: Front Psychol. 2022 Jan 27;12:768616. doi: 10.3389/fpsyg.2021.768616 (PMC8829433; doi:10.3389/fpsyg.2021.768616)
Supplement: Supplementary file 1 [file Data_Sheet_1.docx]

**Multicultural Psychological Empowerment Scale for Saudi Women**

**(MPES - SW)**

| N. | Phrases | Responses | | | | | | | |
| --- | --- | --- | --- | --- | --- | --- | --- | --- | --- |
|  |  | Strongly agree | | Agree | | Indecisive | | Disagree | Strongly disagree |
| **First dimension: Meaningfulness** | | | | | | | | | |
|  | I know the value of the duties I do. | 5 | 4 | | 3 | | 2 | | 1 |
|  | I am aware of the impact of my goals achieving on various aspects of my life. | 5 | 4 | | 3 | | 2 | | 1 |
|  | I realize the usefulness of my community participation. | 5 | 4 | | 3 | | 2 | | 1 |
|  | I take advantage of every opportunity to develop my personality. | 5 | 4 | | 3 | | 2 | | 1 |
| **Second dimension:** **Impact** | | | | | | | | | |
|  | I can influence everyone around me. | 5 | 4 | | 3 | | 2 | | 1 |
|  | I invest my abilities in helping all members of my social context. | 5 | 4 | | 3 | | 2 | | 1 |
|  | I have the ability to persuade others in various situations. | 5 | 4 | | 3 | | 2 | | 1 |
|  | I contribute to creating a positive and moral atmosphere in all around me. | 5 | 4 | | 3 | | 2 | | 1 |
|  | I pass through my thoughts to others. | 5 | 4 | | 3 | | 2 | | 1 |
|  | I take advocacy positions of the rights of others. | 5 | 4 | | 3 | | 2 | | 1 |
|  | I take clear positions on various issues of life. | 5 | 4 | | 3 | | 2 | | 1 |
|  | I make a positive moral difference in my various relationships. | 5 | 4 | | 3 | | 2 | | 1 |
|  | I try to give power to those who need it from my social circle. | 5 | 4 | | 3 | | 2 | | 1 |
|  | I can establish effective alliances with others. | 5 | 4 | | 3 | | 2 | | 1 |
| **Third dimension:** **Self-efficacy** | | | | | | | | | |
|  | I control my behavior in the situations that need it. | 5 | 4 | | 3 | | 2 | | 1 |
|  | I invest the empowerment opportunities in various fields. | 5 | 4 | | 3 | | 2 | | 1 |
|  | I deal effectively with new situations. | 5 | 4 | | 3 | | 2 | | 1 |
|  | I can change my unwanted life statues. | 5 | 4 | | 3 | | 2 | | 1 |
|  | I have good problem-solving skills. | 5 | 4 | | 3 | | 2 | | 1 |
|  | I manage the various types of available time. | 5 | 4 | | 3 | | 2 | | 1 |
|  | I try to gain everything useful that contributes to achieving my goals | 5 | 4 | | 3 | | 2 | | 1 |
| **Fourth dimension:** **Self-determination** | | | | | | | | | |
|  | I take the initiative in various life situations. | 5 | 4 | | 3 | | 2 | | 1 |
|  | I make my decisions with complete independence. | 5 | 4 | | 3 | | 2 | | 1 |
|  | I institute my relationships of my own free will. | 5 | 4 | | 3 | | 2 | | 1 |
|  | I practice freedom of choice in situations that need it. | 5 | 4 | | 3 | | 2 | | 1 |
|  | I take responsibility for my decisions. | 5 | 4 | | 3 | | 2 | | 1 |
